# Impact of accreditation on health care services performance in Kiryandongo district, Uganda: a longitudinal study

**DOI:** 10.1186/s12913-022-07603-4

**Published:** 2022-02-10

**Authors:** Moses Matovu, Elias Musiime, Patrick Olak, Muhammad Mulindwa, Eve Namisango, Kilian Songwe

**Affiliations:** 1 AGHPF, Kampala, Uganda; 2 Kiryandongo District Local Government, Kampala, Uganda

**Keywords:** Quality management systems, Accreditation, SLMTA/SLIPTA

## Abstract

**Background:**

The COVID-19 pandemic has emphasised the need for quality laboratory services worldwide. There is renewed focus to strengthen country capacities and laboratories to effectively respond to public health emergencies and patient outcomes. Uganda launched the accreditation program for public health facilities in 2016 with sixteen laboratories. As of June 2021, twenty-three public laboratories have attained ISO 15189:2012 accreditation status. Despite the tremendous achievements of accrediting laboratories in Uganda, laboratory services still face challenges like stock out of commodities and limited testing scopes. We conducted this study to evaluate the impact of accreditation on health care services performance in *Kiryandongo* district, Uganda.

**Methods:**

We conducted a longitudinal study from January 1, 2020- April 30, 2021 at ten health facilities in *Kiryandongo* district. We collected health care services performance data from the MoH dhis-2 on selected indicators for HIV, TB, Malaria, Laboratory, Maternal & child health and dhis-2 reporting. We used Generalized Estimating Equations to estimate the impact of accreditation on health care services performance at the different health facilities.

**Results:**

The odds at the accredited facility in comparison to the non-accredited public facilities were; 14% higher for ART enrolment (OR = 1.14, 95% CI: 1.04–1.25), 9% lower for determine testing kits stock out (OR = 0.91, 95% CI: 0.85–0.97), 28% higher for TB case diagnosis (OR = 1.28, 95% CI: 1.10–1.49), 19% higher for TB case enrolment (OR = 1.19, 95% CI: 1.04–1.36), 104% higher for maternity admissions (OR = 2.04, 95% CI: 1.60–2.59), 63% higher for maternity deliveries (OR = 1.63, 95% CI: 1.39–1.90) and 17% higher for reporting hmis 10:01 data to dhis-2 (OR = 1.17, 95% CI: 1.04–1.31).

The odds at the accredited facility in comparison to the non-accredited PNFP facilities were; 26% higher for ART enrolment (OR = 1.26, 95% CI: 1.17–1.36), 33% higher for TB case diagnosis (OR = 1.33, 95% CI: 1.15–1.55), 24% higher for TB case enrolment (OR = 1.24, 95% CI: 1.09–1.42), 136% higher for maternity admissions (OR = 2.36, 95% CI: 1.89–2.94), 76% higher for maternity deliveries (OR = 1.76, 95% CI: 1.51–2.04) and 2% higher for reporting of hmis-10:01 data to dhis-2 (OR = 1.02, 95% CI: 1.01–1.03).

**Conclusions:**

HIV, TB, laboratory, MCH, and reporting to dhis-2 selected indicators were positively impacted by accreditation. This impact translated into increased health care services performance at the accredited facility as compared to the non-accredited facilities.

## Background

The COVID-19 pandemic has emphasized the need for improved health care and quality laboratory services worldwide [1]. There is renewed focus to strengthen country capacities and laboratories to promptly and effectively respond to public health risks, emergencies and patient health care [2]. Strengthened laboratory services are vital in the response to public health risks, epidemics, pandemics, health care improvement and attainment of national and international development goals like the UNAIDS 95–95-95 and Sustainable Development Goals [3].

Over the last decade, there has been remarkable improvement in health care performance in resource limited settings due to adoption of quality management systems [4]. The implementation of quality management systems is viewed as a long term, viable, sustainable solution to the vast challenges affecting health services in resource limited settings [5].

Quality management systems like laboratory accreditation have been utilized in developed countries to enhance patient health care and laboratory competencies [6–8]. Accredited systems, provide credible laboratory results, strengthen cross data exchange, decrease costs, wastage and improve health care services performance [9].

Quality Management System initiatives like the Strengthening Laboratory Quality Management Systems Towards Accreditation/Stepwise Laboratory Quality Improvement Process Towards Accreditation (SLMTA/SLIPTA) have been rolled out to many resource limited countries such as Uganda with remarkable results [10, 11].

Uganda adopted the SLMTA/SLIPTA program in 2010 to strengthen the delivery and quality of laboratory services at all health care levels. Over time, laboratories that attained the required performance on the SLMTA/SLIPTA program were supported to attain international accreditation status by the implementing, development partners and Ministry of Health (MoH).

Further on, Uganda launched the accreditation program for public facilities in 2016 with sixteen laboratories. The laboratories were supported through provision of technical assistance that included specialized training, mentorships, evaluations and application for accreditation assessment to the South Africa National Accreditation Service (SANAS). As of June 2021, twenty three (23) public laboratories have attained ISO 15189:2012 accreditation status with more laboratories in the pipeline for international accreditation [12].

Despite the tremendous achievements accrued by the implementation of laboratory quality management system initiatives in Uganda, laboratory services still face challenges including stock out of health commodities, inadequate human resource, limited testing scopes and inadequate funding [13, 14]. These challenges compromise health care performance, and attainment of national and international goals. In addition, health care developments like universal health care coverage, patient centred health care and the ambitious sustainable development health goals are raising the bar for health and laboratory systems to produce better health care performance [15].

There is need to establish impactful health care system models that best suit resource limited settings without compromising the quality of health care and patient safety. Since the establishment, implementation and sustenance of quality management systems such as accreditation entail significant investment, assessing the impact of these systems on health care services is crucial.

We therefore designed this study to evaluate the impact of accreditation on health care services performance at three health facility categories in *Kiryandongo* district, Uganda. The findings from the study inform key stakeholders and MoH on the impact of implementing the accreditation model at the various health facilities in *Kiryandongo* district, Uganda.

## Methods

### Study design

We conducted a longitudinal study from January 1, 2020 to April 30, 2021 at ten health facilities in *Kiryandongo* district, Uganda.

The health facility categories were defined as:

- Accredited public: defined as MoH owned health facility that has attained ISO 15189:2012 status;
- Non-accredited public: defined as MoH owned health facility that has not attained ISO 15189:2012 status;
- Non-accredited PNFP: defined as a private not for profit health facility that has not attained ISO 15189:2012 status.

### Study setting

*Kiryandongo* district is one of the 146 districts in Uganda [16]. The district is found approximately 218 km north of *Kampala* (the capital city of Uganda) in the mid-western part of Uganda (Latitude:2.0000; Longitude:32.3000) at the intersection of Uganda’s prime productive northern and southern regions.

The 2021 population projection by the Uganda Bureau of Statistics (UBOS) recorded *Kiryandongo’s* population at 322,300, of which 163,100 (50.6%) are males and 159,200 (49.4%) are females [17]. *Kiryandongo* district also hosts refugees from South Sudan, internally displaced persons from the Lord’s Resistance Army rebel activities, and person’s displaced by the Eastern region’s *Bududa* landslides of 2010 [18].

The district has thirty-nine (39) health facilities constituting of twenty-one (21) Government, fourteen (14) Private for Profit and four (4)-Private not for profit (PNFP). These facilities serve the population of *Kiryandongo* and the surrounding districts of *Masindi, Nakasongola, Oyam, Apac*, *Amuru* and *Nwoya*. Ten (25.6%) of the health facilities are at Health Center III (HC III) and General Hospital level. The HC IIIs and General Hospitals also provide laboratory services [19].

The General Hospitals offer a variety of services including; Out-patient, Inpatient, Opthalmology, X-ray, Ultra sound, Orthopedics, Health promotion, Health education, Occupational therapy, HIV, Immunization, Environmental health, Special clinics, Support supervision to the lower-level health facilities among other services [19].

*Kiryandongo* General Hospital (KGH) and Restoration Gateway Hospital provide the highest level of health care services within the district. *Kiryandongo* General Hospital has a Biosafety Level II laboratory that has been accredited to ISO 15189:2012 since 2018 (SANAS #: M0624) [20].

The HC IIIs within the district provide basic preventive, promotive, curative care, support supervision of the lower-level facilities under their jurisdiction. The HC IIIs also provide maternal & child care services and first level referral cover at the sub county level [19].

### Study procedure

We selected all health facilities at the HC III and General Hospital level. This was because of the availability of laboratory services at these levels. We collected health care services performance data from the MoH dhis-2 on a quarterly basis on selected HIV, TB, Malaria, Laboratory services, Maternal & child health and reporting to dhis-2 indicators. Data on the baseline characteristics of the health facilities was obtained from the National Health facility Inventory [19]. We used Generalized Estimating Equations to estimate the impact of accreditation on health care services performance at the different health facility categories.

The study was approved by the *Kiryandongo* District Health Team as an exempt study. This was because the study utilized secondary data and did not require the collection of any identifiable data from any participants.

### Study variables

#### Independent variable

The independent variable was accreditation status of the health facility and was categorized as; Accredited public, Non-accredited public and Non-accredited PNFP.

#### Dependent variable

The dependent variable was health care services performance defined as aggregated data on selected HIV, TB, Malaria, Laboratory, Maternal Child & Health and dhis-2 reporting indicators obtained from the MoH dhis-2 system.

We adapted the Global indicator framework for the Sustainable Development Goals and targets of the 2030 Agenda for Sustainable Development and designed health care performance indicators for critical public health concerns in Uganda as shown Table 1 [21]. Health care performance was measured as a non-composite variable for each of the selected indicators.

**Table 1:** Health care services performance and indicators

Health Care Service performance	Indicators
1.)HIV	1.1 Number of new clients enrolled on ART,
	1.2 Days out of stock for ART
	° Tenofovir/Lamivudine/Dolutegravir (TDF/3TC/DTG),
	° Abacavir/Lamivudine (ABC/3TC)],
	1.3 Days out of stock for HIV testing kits
	° Determine test kits,
	° Sat Pak test kits
2.)TB	2.1 Number of TB cases identified,
	2.2 Number of TB cases diagnosed;
	2.3 Number of TB cases enrolled
	2.4 Days out of stock for GeneXpert cartridges
3.)Malaria	3.1 Number of malaria cases identified
	3.2 Number of malaria cases diagnosed
4.)Maternal and Child health	4.1Number of maternity admissions,
	4.2 Number of maternity deliveries,
	4.3 Number of BCG immunisations administered
	4.4Number of Pentavalent immunisations administered
	4.5Number of children dewormed
5.)Laboratory services	5.1 Number of samples referred to the laboratory,
	5.2 Number of Hepatitis B samples tested
6.)Reporting to dhis-21.)	6.1 Percentage of Hmis 10: 01 data (attendances, referrals, TB) reported,
	6.2 Percentage of hmis 10:10 data (laboratory) reported

### Statistical analysis

Descriptive analysis was undertaken to profile the health facilities by level of service delivery, type of ownership, accreditation status and catchment population. Categorical variables were summarised by percentage. Continuous variables were summarised by median and inter quartile range (IQR).

Prior to analysis, all data were tested for normality using the Shapiro-Wilk test and for homogeneity of variances by Levene’s test. To assess differences in health care services performance across and between the health facility categories, non-parametric tests, Kruskal-Wallis and Mann-Whitney test were performed. Performance was considered significant at *p* < 0.05.

We used a Generalized Estimating Equation (GEE) model to model the quarterly clustered data within each health facility and estimated the impact of accreditation on the health care services performance at the health facility categories.

## Results

### Baseline characteristics

A total of ten facilities (10) participated in the study. Table 2 describes the baseline characteristics of the facilities.

**Table 2:** Baseline characteristics of study facilities

Baseline characteristic	Accredited *n* = 1		Non-Accredited public *n* = 5		Non- Accredited PNFP *n* = 4	
	Number	Percentage	Number	Percentage	Number	Percentage
Total facilities	1	10%	5	50%	4	40%
Accreditation (ISO 15189: 2012)						
Accredited	1	10%	0	0%	0	0%
Not Accredited	0	0%	5	50%	4	40%
Health Service delivery level						
General Hospital	1	10%	0	0%	1	10%
HC III	0	0%	5	50%	3	30%
Ownership						
Public	1	10%	5	50%	0	0%
PNFP	0	0%	0	0%	4	40%
Catchment population						
20,000	0	0%	5	50%	3	30%
500,000	1	10%	0	0%	1	10%

*PNFP* Private not for profit

By level of health service delivery 80% (8/10) of the facilities were HC III. By type of ownership, 60% (6/10) of the facilities were public, 90% (9/10) of the facilities were not accredited and 20% (2/10) of the facilities had a catchment population of 500,000 people.

### Health care services performance

We used the Kruskal-Wallis tests to assess the differences in the health care services performance across the health facility categories. Performance was considered significant at *p* < 0.05. Table 3 describes the comparison of the health care services performance across the health facility categories.

**Table 3:** Comparison of healthcare services performance at Accredited, Non-Accredited public and Non-Accredited PNFP health facilities

Healthcare Services Performance, (Median, IQR)	Accredited *n* = 1 (10%)	Non-Accredited Public *n* = 5 (50%)	Non-Accredited PNFP *n* = 4 (40%)	Kruskal-Wallis (H)	*P*-Value
HIV					
New clients on ART per 100 pop	0.34 (0.33–0.39)	0.19 (0.12–0.26)	0.13 (0.09–0.18)	12.954	0.002*
DOS-TDF/3TC/DTG	3.00 (1.00–16.00)	0.00 (0.00–0.00)	0.00 (0.00–0.00)	8.720	0.013*
DOS-ABC/3TC	1.00 (0.00–14.00)	0.00 (0.00–0.000)	0.00 (00.0–0.00)	13.535	0.001*
DOS-determine test kits	0.00 (0.00–0.00)	0.00 (0.00–11.00)	0.00 (0.00–0.00)	6.407	0.041*
DOS-Stat Pak test kits	0.00 (0.00–0.00)	0.0 (0.00–30.00)	0.00 (0.00–0.00)	3.541	0.170
TB					
Cases identified per 100 pop	0.33 (0.18–0.48)	0.17 (0.09–0.25)	0.04 (0.01–0.12)	14.338	0.001*
Cases diagnosed per 100 pop	0.30 (0.20–0.36)	0.04 (0.03–0.08)	0.02 (0.01–0.02)	22.207	0.000*
Cases enrolled per 100 pop	0.20 (0.09–0.41)	0.04 (0.03–0.10)	0.01 (0.01–0.03)	16.480	0.001*
DOS-GeneXpert cartridges	0.00 (0.00–30.00)	31.00 (30.00–60.00)	0.00 (0.00–15.00)	5.961	0.051
Malaria per 1000 pop					
Cases identified	1.16 (0.64–1.43)	1.16 (0.62–2.13)	0.23 (0.13–0.36)	27.141	0.000*
Cases diagnosed	1.11 (0.60–1.37)	1.07 (0.62–2.02)	0.22 (0.13–0.36)	27.419	0.000*
Laboratory Services per 100 pop					
Referral samples	5.01 (3.18–7.76)	0.29 (0.18–0.67)	0.67 (0.35–1.72)	5.426	0.066
Hep B Testing	3.21 (2.21–5.80)	3.45 (0.35–5.16)	0.88 (0.54–1.43)	3.919	0.141
MCH services per 1000 pop					
Maternity Admissions	1.08 (1.06–1.13)	0.17 (0.09–0.21)	0.12 (0.10–0.14)	19.013	0.000*
Maternity deliveries	0.73 (0.69–0.77)	0.15 (0.07–0.17)	0.09 (0.06–0.11)	22.154	0.000*
BCG immunisation	0.86 (0.79–0.87)	0.24 (0.09–0.28)	0.09 (0.07–0.15)	18.888	0.000*
Pentavalent immunisation	0.42 (0.38–0.45)	0.23 (0.09–0.27)	0.08 (0.05–0.19)	17.332	0.000*
Deworming	0.26 (0.25–0.67)	0.56 (0.09–2.51)	0.11 (0.02–0.59)	3.559	0.169
Reporting to dhis-2					
hmis 105:01 (attendances, referrals)	61.15 (47.20–66.7)	33.30 (33.30–33.30)	33.30 (33.30–33.30)	20.206	0.000*
hmis 105:10 (laboratory)	38.85 (24.98–44.40)	33.30 (33.00–33.00)	33.30 (33.30–33.30)	4.030	0.133

*Pop* Population, *DOS* Days out of stock, *PNFP* Private Not for Profit

### Sub group analysis: comparison between health categories

We conducted a sub-group analysis and compared the differences in health care performance between the health facility categories using the Mann-Whitney test. The results from the sub group analysis are shown in Table 4.

**Table 4:** Comparison of healthcare Services performance at Accredited, Non-Accredited public and Non-Accredited PNFP health facilities

Healthcare Services Performance (Median, IQR)	Accredited *n* = 1 (10%)	Non-Accredited Public *n* = 5 (50%)	*P*-Value	Accredited *n* = 1 (10%)	Non-Accredited PNFP *n* = 4 (40%)	*P*-Value	Non-Accredited Public *n* = 5 (50%)	Non-Accredited PNFP *n* = 4 (40%)	*P*-Value
HIV									
New clients on ART per 100 pop	0.34 (0.33–0.39)	0.19 (0.12–0.26)	0.028*	0.34 (0.33–0.39)	0.13 (0.09–0.18)	0.001*	0.19 (0.12–0.26)	0.13 (0.09–0.18)	0.025*
DOS-TDF/3TC/DTG	3.00 (1.00–16.00)	0.00 (0.00–0.00)	0.085	3.00 (1.00–16.00)	0.00 (0.00–0.00)	0.002*	0.00 (0.00–0.00)	0.00 (0.00–0.00)	0.177
DOS-ABC/3TC	1.00 (0.00–14.00)	0.00 (0.00–0.000)	0.010*	1.00 (0.00–14.00)	0.00 (00.0–0.00)	0.001*	0.00 (0.00–0.000)	0.00 (00.0–0.00)	0.218
DOS-determine test kits	0.00 (0.00–0.00)	0.00 (0.00–11.00)	0.143	0.00 (0.00–0.00)	0.00 (0.00–0.00)	0.511	0.00 (0.00–11.00)	0.00 (0.00–0.00)	0.028*
DOS-Stat Pak test kits	0.00 (0.00–0.00)	0.0 (0.00–30.00)	0.186	0.00 (0.00–0.00)	0.00 (0.00–0.00)	0.402	0.0 (0.00–30.00)	0.00 (0.00–0.00)	0.138
TB									
Cases identified per 100 pop	0.33 (0.18–0.48)	0.17 (0.09–0.25)	0.132	0.33 (0.18–0.48)	0.04 (0.01–0.12)	0.006*	0.17 (0.09–0.25)	0.04 (0.01–0.12)	0.001*
Cases diagnosed per 100 pop	0.30 (0.20–0.36)	0.04 (0.03–0.08)	0.001*	0.30 (0.20–0.36)	0.02 (0.01–0.02)	0.001*	0.04 (0.03–0.08)	0.02 (0.01–0.02)	0.000*
Cases enrolled per 100 pop	0.20 (0.09–0.41)	0.04 (0.03–0.10)	0.009*	0.20 (0.09–0.41)	0.01 (0.01–0.03)	0.001*	0.04 (0.03–0.10)	0.01 (0.01–0.03)	0.003*
DOS-GeneXpert cartridges	0.00 (0.00–30.00)	31.00 (30.00–60.00)	0.066	0.00 (0.00–30.00)	0.00 (0.00–15.00)	0.731	31.00 (30.00–60.00)	0.00 (0.00–15.00)	0.059
Malaria per 100 pop									
Cases identified	1.16 (0.64–1.43)	1.16 (0.62–2.13)	0.552	1.16 (0.64–1.43)	0.23 (0.13–0.36)	0.005*	1.16 (0.62–2.13)	0.23 (0.13–0.36)	0.000*
Cases diagnosed	1.11 (0.60–1.37)	1.07 (0.62–2.02)	0.511	1.11 (0.60–1.37)	0.22 (0.13–0.36)	0.005*	1.07 (0.62–2.02)	0.22 (0.13–0.36)	0.000*
Laboratory Services per 100 pop									
Referral samples	5.01 (3.18–7.76)	0.29 (0.18–0.67)	0.050	5.01 (3.18–7.76)	0.67 (0.35–1.72)	0.041*	0.29 (0.18–0.67)	0.67 (0.35–1.72)	0.540
Hep B Testing	3.21 (2.21–5.80)	3.45 (0.35–5.16)	0.737	3.21 (2.21–5.80)	0.88 (0.54–1.43)	0.081	3.45 (0.35–5.16)	0.88 (0.54–1.43)	0.121
MCH services per 1000 pop									
Maternity Admissions	1.08 (1.06–1.13)	0.17 (0.09–0.21)	0.000*	1.08 (1.06–1.13)	0.12 (0.10–0.14)	0.000*	0.17 (0.09–0.21)	0.12 (0.10–0.14)	0.028*
Maternity deliveries	0.73 (0.69–0.77)	0.15 (0.07–0.17)	0.000*	0.73 (0.69–0.77)	0.09 (0.06–0.11)	0.000*	0.15 (0.07–0.17)	0.09 (0.06–0.11)	0.003*
BCG immunisation	0.86 (0.79–0.87)	0.24 (0.09–0.28)	0.001*	0.86 (0.79–0.87)	0.09 (0.07–0.15)	0.001*	0.24 (0.09–0.28)	0.09 (0.07–0.15)	0.006*
Pentavalent immunisation	0.42 (0.38–0.45)	0.23 (0.09–0.27)	0.020*	0.42 (0.38–0.45)	0.08 (0.05–0.19)	0.001*	0.23 (0.09–0.27)	0.08 (0.05–0.19)	0.002*
Deworming	0.26 (0.25–0.67)	0.56 (0.09–2.51)	0.770	0.26 (0.25–0.67)	0.11 (0.02–0.59)	0.221	0.56 (0.09–2.51)	0.11 (0.02–0.59)	0.081
Healthcare Services Performance (Median, IQR)	Accredited (*n* = 1)	Non-Accredited Public (*n* = 5)	*P*-Value	Accredited (*n* = 1)	Non-Accredited PNFP (*n* = 4)	*P*-Value	Non-Accredited Public (*n* = 5)	Non-Accredited PNFP (*n* = 4)	*P*-Value
Reporting to dhis-2									
hmis 105:01 (attendances, referrals)	61.15 (47.20–66.7)	33.30 (33.30–33.30)	0.008*	61.15 (47.20–66.7)	33.30 (33.30–33.30)	0.000*	33.30 (33.30–33.30)	33.30 (33.30–33.30)	0.007*
hmis 105:10 (laboratory)	38.85 (24.98–44.40)	33.30 (33.00–33.00)	0.980	38.85 (24.98–44.40)	33.30 (33.30–33.30)	0.294	33.30 (33.00–33.00)	33.30 (33.30–33.30)	0.025*

*Pop* Population, *DOS* Days out of stock, *PNFP* Private Not for Profit

### Impact of accreditation on health care services performance

We used Generalized Estimating Equations (GEE) modelling to estimate the impact of accreditation on the health care services performance at the different health facility categories. We selected the health care services performance indicators which achieved a statistical significance of *P* ≤ 0.2 at the bivariate level (Table 2) as the outcome variables. The categorical independent variable was selected as the factor and the health facility catchment population as the covariate. A cut off *P*-value of < 0.05 was adopted for statistical significance. The results from the GEE analysis are shown in Table 5.

**Table 5:** Impact of accreditation on health care services performance at Accredited, Non-Accredited public and Non-Accredited PNFP health facilities

Healthcare services performance	Non-Accredited Public *n* = 5, (50%)	Accredited *n* = 1, (10%)		Non-Accredited PNFP *n* = 4, (40%)	Accredited *n* = 1, (10%)	
	OR	OR (95% CI)	***P***-Value	OR	OR (95% CI)	***P***-Value
HIV						
New clients on ART	1.0	1.14 (1.04–1.25)	0.007*	1.0	1.26 (1.17–1.36)	0.000*
DOS-TDF/3TC/DTG	1.0	1.05 (0.93–1.17)	0.427	1.0	1.06 (0.98–1.21)	0.130
DOS-ABC/3TC	1.0	1.07 (0.97–1.17)	0.171	1.0	1.08 (0.99–1.18)	0.074
DOS-determine test kits	1.0	0.91 (0.85–0.97)	0.008*	1.0	1.01 (0.99–1.01)	0.675
DOS-Stat Pak test kits	1.0	0.87 (0.79–0.96)	0.006*	1.0	1.01 (0.97–1.02)	0.484
TB						
Cases identified	1.0	1.06 (0.62–1.82)	0.836	1.0	1.47 (1.01–2.12)	0.043*
Cases diagnosed	1.0	1.28 (1.10–1.49)	0.001*	1.0	1.33 (1.15–1.55)	0.000*
Cases enrolled	1.0	1.19 (1.04–1.36)	0.010*	1.0	1.24 (1.09–1.42)	0.001*
DOS-GeneXpert cartridges	1.0	0.78 (0.66–0.93)	0.006*	1.0	1.03 (0.83–1.27)	0.795
Malaria						
Cases identified	1.0	0.62 (0.27–1.38)	0.239	1.0	2.16 (1.46–3.21)	0.000*
Cases diagnosed	1.0	0.46 (0.22–0.94)	0.033*	1.0	2.07 (1.41–3.04)	0.000*
Laboratory Services						
Referral samples	1.0	1.67 (1.09–2.57)	0.018*	1.0	1.67 (1.09–2.56)	0.018*
Hep B Testing	1.0	1.04 (0.71–1.54)	0.823	1.0	1.24 (0.86–1.78)	0.254
MCH Services						
Maternity Admissions	1.0	2.04 (1.60–2.59)	0.000*	1.0	2.36 (1.89–2.94)	0.000*
Maternity deliveries	1.0	1.63 (1.39–1.90)	0.000*	1.0	1.76 (1.51–2.04)	0.000*
BCG immunisation	1.0	1.66 (1.38–1.99)	0.000*	1.0	1.79 (1.48–2.17)	0.000*
Pentavalent immunisation	1.0	1.18 (1.06–1.30)	0.002*	1.0	1.26 (1.15–1.39)	0.000*
Reporting to dhis-2						
hmis 105:01 (attendances, referrals)	1.0	1.17 (1.04–1.31)	0.007*	1.0	1.02 (1.01–1.03)	0.000*
hmis 105:10 (laboratory)	1.0	0.74 (0.13–4.21)	0.738	1.0	1.04 (0.88–1.23)	0.668

*PNFP* Private Not for Profit

## Discussions

This study examined the impact of accreditation on health care performance at three health facility categories in *Kiryandongo* district, Uganda.

Accreditation positively impacted the selected indicators; new clients on ART, TB case diagnosis, TB case enrolment, malaria case diagnosis, laboratory sample referral, maternity admissions, maternity deliveries, immunisations and reporting to dhis-2 for hmis 105:10.

Accreditation did not however impact the indicators for; days out of stock for ART medication (TDF/3TC/DTG, ABC/3TC) and Hepatitis B Testing.

### HIV services performance

The accredited facility was more likely to enrol new HIV patients on ART (OR = 1.14) in comparison to the non-accredited public facility and (OR = 1.26) in comparison to the non-accredited PNFP. Furthermore, the accredited facility was less likely to experience stockouts for HIV testing kits (OR = 0.91- Determine, OR = 0.87-StatPak) in comparison to the non-accredited public facility. The difference in new enrolments for HIV and days out of stock for HIV testing kits at the accredited facility were statistically significant, *P* < 0.05. The accredited facility however had higher stockouts for antiretroviral medication (OR = 1.05-TDF/3TC/DTG, OR = 1.07- ABC/3TC). The difference in stockout of antiretroviral medication at the accredited facility was however not statistically significant, *P* > 0.05.

The HIV performance at the accredited facility can be explained by the increased community awareness of the facility’s accreditation status and improved stock management of the HIV testing supplies by the laboratory and the facility. This may have resulted into an increased utilization of the HIV services at the facility. These results are similar to those obtained by Ebong et al., in Cameroon who reported customer satisfaction of up to 64% for the adult volunteers attending HIV Infection Testing and Service Delivery at an accredited treatment Centre [22]. A similar study by Billong et al., in Cameroon, reported an increase in HIV testing performance (sensitivity by 10.2% and specificity by 0.3%) following implementation of quality systems interventions in PMTCT programs [23].

### TB services performance

The accredited facility was more likely to identify, diagnose and enrol TB cases (OR = 1.06, 1.28, 1.19 respectively) compared to the non-accredited public facilities. Similarly, the accredited facility was more likely to identify, diagnose and enrol TB cases (OR = 1.47, 1.33, 1.24 respectively) compared to the non-accredited PNFP facilities. The difference in TB case diagnosis and enrolments at the accredited facility were statistically significant, *P* < 0.05. Further on, the accredited facility had lower stockouts for GeneXpert cartridges (OR = 0.78) compared to the non-accredited public facilities. The difference in days out of stock for TB GeneXpert cartridges at the accredited facility was statistically significant, *P* < 0.05.

The TB services performance at the accredited facility may be explained by the improved stock management, reduced reagent wastage and reduced service interruptions. In addition, the implementation of the National TB program activities such as support supervision by the district health teams, weekly reporting, blinded rechecking and GeneXpert external quality assurance within *Kiryandongo* district may also have contributed to this performance [24]. The National TB program activities aim at strengthening the implementation of TB activities in *Kiryandongo* district and the country at large.

Our findings are similar to those by Getahun et al., in Ethiopia who reported establishment of an effective inventory management system that enabled laboratory supplies forecast, reduced reagent wastage and service interruptions to tuberculosis direct sputum smear microscopy services. A similar study by Susanne Homolka et al., in Germany reported an increase in odds ratio by 108% for a sample to be tested as microscopically positive following implementation of Quality Management Systems [25, 26]. Furthermore, a study by Brugueras et al. in Spain reported significant differences in TB treatment and management for sample collection, documentation of final results, management of resistance, coordination with other departments, contact tracing and directly observed treatment for accredited TB units when compared to the non-accredited units [27].

### Malaria services performance

The accredited facility identified and diagnosed less malaria cases (OR = 0.62, 0.46 respectively) compared to the non-accredited public facilities. The accredited facility however identified and diagnosed more malaria cases than the non-accredited PNFP facility (OR = 2.16, 2.07 respectively). The difference in malaria identification and diagnosis at the accredited facility was statistically significant, *P* < 0.05.

The findings for malaria performance at the non-accredited public facilities are in tandem with the role played by the HCIIIs as the first level referral cover for laboratory testing in the district. This is aimed at preventing decongestion at the higher levels of health care by the common conditions in the district. The findings at the non-accredited PNFPs may also be explained by the nominal fees being charged for malaria testing whereas in the public facilities, testing is paid for by the MoH hence free to the public.

The Malaria performance findings emphasize the need to implement quality management systems at the lower health facilities that constitute a large bulk of malaria treatment. Our findings are in tandem with those obtained by Thomson et al., in Tanzania [28] and Dorkenoo et al., who showed that the implementation of quality management systems, refresher training and expanded PT at remote testing facilities are essential for improving the quality of malaria diagnosis [29].

### Laboratory services performance

The accredited facility was also more likely to receive referral samples (OR = 1.67) as compared to the non-accredited public and PNFP facilities. The difference in sample referral to the accredited facility was statistically significant, *P* < 0.05. This finding may be explained by the designation of the accredited facility as a hub for the district by MoH. The facilities within the district are mandated to refer samples for viral load testing, early infant diagnosis among other tests to the National Reference Laboratories through the hub system [30].

Further on, the accredited facility was more likely to conduct Hepatitis B testing compared to the non-accredited public and PNFP facilities (OR = 1.04, 1.24 respectively). The difference in Hepatitis B testing at the accredited facility was however not statistically significant, *p* > 0.05. This finding may be due to the implementation of the National Hepatitis B activities such as free testing, mass vaccination campaigns conducted at the different health facilities in the district. These activities are in line with MoH’s agenda of advancing the fight against Hepatitis B disease [31].

### Maternal and child health services

The accredited facility was more likely to register maternity admissions, maternity deliveries, BCG immunisations and pentavalent immunisation (OR = 2.04, 1.63, 1.66, 1.18 respectively) compared to the non-accredited public facilities.

Similarly, the accredited facility was more likely to register maternity admissions, maternity deliveries, BCG immunisations and pentavalent immunisation (OR = 2.36, 1.76, 1.79, 1.26 respectively) compared to the non-accredited PNFP facilities. The difference in maternity admissions, maternity deliveries, BCG immunisations and pentavalent immunisation at the accredited facility were statistically significant, *p* < 0.05.

The maternal performance at the accredited facility may be explained by the increased community awareness of accreditation of the facility that resulted into increased utilization of the facility maternity and child health services. Our findings are in tandem with the results by El-Shal et al., who showed that accreditation in Egypt was associated with significant improvements in child morbidity, family planning, and delivery care [7]. Similarly, Chao-Wen et al., in South West China, reported improvements in in a new born screening program for indicators including new born health education provision, dried blood sampling, turnaround time, new born recall after positive primary screening following implementation of a comprehensive quality management system [32].

### Reporting to dhis-2 performance

The accredited facility was more likely to report to dhis-2, data for hmis 10:01 (attendances, referrals) compared to the non-accredited public and PNFP facilities (OR = 1.17, 1.02 respectively). The difference in reporting for hmis 10:01 data at the accredited facility was statistically significant, *p* < 0.05.

In addition, the accredited facility was more likely to report to dhis-2, data for hmis 10:10 (laboratory) compared to the non-accredited PNFP facilities (OR = 1.04) and less likely compared to the non-accredited public facilities (OR = 0.74). The difference in reporting for hmis 10:10 data at the accredited facility was however not statistically significant, *p* > 0.05.

The reporting to dhis-2 performance may be explained by MoH requirement to the health facilities to report health care services performance through the dhis-2 system. The performance for reporting at the accredited facility may also be explained by the improved records & data management and compliance to the reporting standard operating procedures by MoH.

Our findings are in tandem with the results by Maddux et al., who showed increased compliance by 22% with reporting standards for accredited laboratories [33]. A similar study by Kibet et al., reported improvement in critical value reporting by 33 and 40% improvement for laboratory Turn Around Time following accreditation [34].

Results of this study complement what is stated within the literature about the positive impact of accreditation on quality improvement [35–40]. A systematic review that examined different aspects of the hospitals like bed sizes, geographic regions, teaching status, ownership, disease areas and service types reported that accreditation may have a positive impact on efficiency, safety, effectiveness, timeliness and patient-centeredness [41].

Whereas the benefits of accreditation are well documented, implementation and compliance to accreditation requirements are challenging and demanding, requiring active involvement from all key stakeholders to sustain the accreditation status [42, 43]. However, the investment in accreditation yields positive returns as demonstrated in this study.

This study had limitations including utilization of secondary data, as a result we could not eliminate bias from unobserved variables about the health facilities. A follow-up study with a more robust design, such as prospective cohort, should be conducted. In addition, the study was conducted within the period of the COVID-19 pandemic which also included the national lockdown. The lock down restricted patient movements, and impacted logistics and supply chain mechanisms which could have affected stock availability at the respective health facilities. It is possible that the health performance could have been higher than what was eventually captured.

## Conclusions

HIV, TB, Laboratory services, Maternal & child health and reporting to dhis-2 performance indicators were positively impacted by accreditation. This impact translated into increased health care performance at the accredited facility compared to the non-accredited facilities.

The attainment of accreditation comes with several requirements which may include facility upgrades, building personnel capacity, establishing functional equipment management programs and participation in external quality assurance programs. These requirements often form barriers for resource limited settings to realize accreditation, especially if the value for investment is not demonstrated. Accreditation programs of health laboratories should however be encouraged and supported to improve the standard of health care services and patient safety given the positive impact demonstrated by this study.

## Data Availability

The datasets used and/or analyzed during the study are available from the corresponding author and can be provided on request.

## Abbreviations

ABC/3TC: Abacavir/Lamivudine
ART: Anti-Retroviral Theraphy
BCG: Bacillus Calmette–Guérin
COVID-19: Corona Virus Disease 2019
DOS: Days out of stock
HC III: Health Center III
HMIS: Health Management Information System
ISO: International Organization for Standardization
MCH: Maternal and Child Health Services
MoH: Ministry of Health
PNFP: Private not for profits
Pop: Population
SANAS: South Africa National Accreditation Service
SLIPTA: Stepwise Laboratory Quality Improvement Process Toward Accreditation
SLMTA: Strengthening Laboratory Quality Management Systems towards Accreditation
TDF/3TC/DTG: Tenofovir/Lamivudine/Dolutegravir
UBOS: Uganda Bureau of Statistics
UNAIDS: United Nations Programme on HIV/AIDS
WHO: World Health Organization

## References

[1] Pawar SD, Kode SS, Keng SS, Tare DS, Abraham P (2020). Steps, implementation and importance of quality management in diagnostic laboratories with special emphasis on coronavirus disease-2019. Indian J Med Microbiol.

[2] Subbian V, Solomonides A, Clarkson M, Rahimzadeh VN, Petersen C, Schreiber R, DeMuro PR, Dua P, Goodman KW, Kaplan B (2021). Ethics and informatics in the age of COVID-19: challenges and recommendations for public health organization and public policy. J Am Med Inform Assoc.

[3] Adane K, Girma M, Deress T. How does ISO 15189 laboratory accreditation support the delivery of healthcare in Ethiopia? A systematic review. Ethiop J Health Sci. 2019;29(2). https://www.ajol.info/index.php/ejhs/article/view/188125. 10.4314/ejhs.v29i2.13 PMC6460445 31011274

[4] Yao K, Luman ET (2016). Evidence from 617 laboratories in 47 countries for SLMTA-driven improvement in quality management systems. Afr J lab Med.

[5] Kruk ME, Gage AD, Arsenault C, Jordan K, Leslie HH, Roder-DeWan S, Adeyi O, Barker P, Daelmans B, Doubova SV (2018). High-quality health systems in the sustainable development goals era: time for a revolution. Lancet Glob Health.

[6] Rusanganwa V, Gahutu JB, Evander M, Hurtig A-K (2019). Clinical referral laboratory personnel’s perception of challenges and strategies for sustaining the laboratory quality management system: a qualitative study in Rwanda. Am J Clin Pathol.

[7] El-Shal A, Cubi-Molla P, Jofre-Bonet M (2021). Accreditation as a quality-improving policy tool: family planning, maternal health, and child health in Egypt. Eur J Health Econ.

[8] Zhai P, Wang R, Zhou Y, Hu D, Zhou Y (2019). Enhancing the capabilities of biosafety laboratories through the established accreditation system: development of the biosafety laboratory accreditation system in China. J Biosafety Biosecur.

[9] Shpilsky D, Harinstein ME. Evaluation of the impact of laboratory accreditation on downstream outcomes. J Nucl Cardiol. 2021;28(6):2962–4. https://link.springer.com/article/10.1007/s12350-020-02292-0. 10.1007/s12350-020-02292-0 32715417

[10] Andiric LR, Chavez LA, Johnson M, Landgraf K, Milner DA (2018). Strengthening laboratory management toward accreditation, a model program for pathology laboratory improvement. Clin Lab Med.

[11] Sisay A, Gurmessa A, Liknew W (2019). Factors affecting implementation of laboratory quality management system in Addis Ababa public health laboratories, Addis Ababa, Ethiopia. J Trop Dis Public Health.

[12] SANAS. South Africa national accreditation system: conformity assessment bodies (CAB). Uganda: South Africa National Accreditation Systems; 2021. https://www.sanas.co.za/Pages/index.aspx.

[13] Nalugwa T, Shete PB, Nantale M, Farr K, Ojok C, Ochom E, Mugabe F, Joloba M, Dowdy DW, Moore DA (2020). Challenges with scale-up of GeneXpert MTB/RIF® in Uganda: a health systems perspective. BMC Health Serv Res.

[14] Ackers L, Ackers-Johnson G, Seekles M, Odur J, Opio S (2020). Opportunities and challenges for improving anti-microbial stewardship in low-and middle-income countries; lessons learnt from the maternal sepsis intervention in western Uganda. Antibiotics.

[15] Tashkandi SA, Alenezi A, Bakhsh I, AlJuryyan A, AlShehry ZH, AlRashdi S, Guzman M, Pono M, Lim F, Tabudlong AR (2021). Clinical laboratory services for primary healthcare centers in urban cities: a pilot ACO model of ten primary healthcare centers. BMC Fam Pract.

[16] GOU (2021). Government of Uganda, Electoral Commission Statistics.

[17] UBOS (2021). Uganda Bureau of Statistics, Population and Censuses.

[18] UIA: Uganda Investment Authority, Kiryandongo district: Investment profile. n.d.

[19] MoH (2018). Ministry of Health.

[20] SANAS (2021). South Africa national accreditation systems: conformity assessment bodies: kiryandongo general hospital laboratory.

[21] UN (2021). United Nations, SDG Indicators: Global indicator framework for the sustainable development goals and targets of the 2030 Agenda for Sustainable Development.

[22] Ebong SB, Penda CI, Enoné JPM, Eboumbou PE, Mbangue M, Mandengue SH, Moukoko CEE (2020). HIV infection testing and service delivery of one accredited treatment Center in Cameroon. Int J HIV/AIDS Prev Educ Behav Sci.

[23] Billong SC, Fokam J, Ndé CK, Penda I, Messeh A, de Dieu AJ, et al. Decreased inaccuracies in HIV screening following strengthening of quality system: evidence-based interventions through the PMTCT program in Cameroon. Health Sci Dis. 2019;20(2).

[24] MoH (2020). Ministry of Health, Uganda, Uganda National TB and Leprosy Program.

[25] Getahun MS, Yemanebrhane N, Desalegn DM, Kitila KT, Dinku TT, Wondimagegnehu DD, Wolde EA, Bika AT, Mersha TB, Asferie KD (2019). Medical laboratory accreditation in a resource-limited district health Centre laboratory, Addis Ababa Ethiopia. Afr J lab Med.

[26] Homolka S, Zallet J, Albert H, Witt A-K, Kranzer K (2019). Introduction of quality management in a National Reference Laboratory in Germany. PLoS One.

[27] Brugueras S, Roldán L, Rodrigo T, García-García J-M, Caylà JA, García-Pérez FJ, Orcau À, Viladrich IM, Penas-Truque A, Millet J-P (2020). Organization of tuberculosis control in Spain: evaluation of a strategy aimed at promoting the accreditation of tuberculosis units. Arch Bronconeumol (Engl Ed).

[28] Thomson R, Johanes B, Festo C, Kalolella A, Taylor M, Tougher S, Ye Y, Mann A, Ren R, Bruxvoort K (2018). An assessment of the malaria-related knowledge and practices of Tanzania’s drug retailers: exploring the impact of drug store accreditation. BMC Health Serv Res.

[29] Dorkenoo AM, Kouassi KC, Koura AK, Adams ML, Gbada K, Katawa G, Yakpa K, Charlebois R, Milgotina E, Merkel MO (2021). The use of dried tube specimens of plasmodium falciparum in an external quality assessment programme to evaluate health worker performance for malaria rapid diagnostic testing in healthcare centres in Togo. Malar J.

[30] MoH (2017). Guidelines for the Uganda National Health Laboratory hub andSample transport network.

[31] Uganda Commended for Efforts in the fight against Hepatitis B [https://www.health.go.ug/2019/12/02/uganda-commended-for-efforts-in-the-fight-against-hepatitis-b/].

[32] Yu C-W, He X-Y, Wan K-X, Yuan Z-J, Liu H, Zhang J, Liu S, Yang J, Zou L (2021). Improving quality management of newborn screening in Southwest China. J Int Med Res.

[33] Maddux PT, Farrell MB, Ewing JA, Tilkemeier PL (2018). Improved compliance with reporting standards: a retrospective analysis of Intersocietal accreditation commission nuclear cardiology laboratories. J Nucl Cardiol.

[34] Kibet E, Moloo Z, Ojwang PJ, Sayed S, Mbuthia A, Adam RD (2014). Measurement of improvement achieved by participation in international laboratory accreditation in sub-Saharan Africa: the Aga Khan University hospital Nairobi experience. Am J Clin Pathol.

[35] Reisi N, Raeissi P, Sokhanvar M, Kakemam E (2019). The impact of accreditation on nurses' perceptions of quality of care in Iran and its barriers and facilitators. Int J Health Plann Manag.

[36] Ghareeb A, Said H, El Zoghbi M (2018). Examining the impact of accreditation on a primary healthcare organization in Qatar. BMC Med Educ.

[37] Tabrizi JS, Gharibi F. Primary healthcare accreditation standards: a systematic review. Int J Health Care Qual Assur. 2019. http://hsd-fmsb.org/index.php/hsd/article/view/1264. 10.1108/IJHCQA-02-2018-0052 31017069

[38] Andres EB, Song W, Song W, Johnston JM (2019). Can hospital accreditation enhance patient experience? Longitudinal evidence from a Hong Kong hospital patient experience survey. BMC Health Serv Res.

[39] Avia I, RTSJEC H (2019). Impact of hospital accreditation on quality of care: a literature review. Enferm Clin.

[40] Nicklin W, Fortune T, van Ostenberg P, O'Connor E, McCauley N (2017). Leveraging the full value and impact of accreditation. Int J Qual Health Care.

[41] Araujo CA, Siqueira MM, Malik AM (2020). Hospital accreditation impact on healthcare quality dimensions: a systematic review. Int J Qual Health Care.

[42] Lapić I, Rogić D, Ivić M, Tomičević M, Kardum Paro MM, Đerek L, Alpeza Viman I (2021). Laboratory professionals’ attitudes towards ISO 15189: 2012 accreditation: an anonymous survey of three Croatian accredited medical laboratories. Biochem Med.

[43] Gopolang F, Zulu-Mwamba F, Nsama D, Kruuner A, Nsofwa D, Kasvosve I, et al. Improving laboratory quality and capacity through leadership and management training: Lessons from Zambia 2016–2018. Afr J Lab Med. 2021;10(1). 10.4102/ajlm.v10i1.1225 PMC8111616 34007816

